# Intelligent Glasses, Watches and Vests…Oh My! Rethinking the Meaning of “Harm” in the Age of Wearable Technologies

**DOI:** 10.2196/mhealth.3565

**Published:** 2015-02-05

**Authors:** Alejandro R Jadad, Marcela Fandiño, Robin Lennox

**Affiliations:** ^1^Centre for Global eHealth InnovationUniversity Health NetworkUniversity of TorontoToronto, ONCanada; ^2^University of British ColumbiaVancouver, BCCanada; ^3^McMaster UniversityFaculty of Health SciencesHamilton, ONCanada

**Keywords:** harm, digital telecommunication technology, wearable computing, Internet, conceptualization

## Abstract

The widespread release and adoption of wearable devices will likely accelerate the “hybrid era”, already initiated by mobile digital devices, with progressively deeper levels of human-technology co-evolution and increasing blurring of our boundaries with machines. Questions about the potentially harmful nature of information and communication technologies have been asked before, since the introduction of the telephone, the Web, and more recently, mobile phones. Our capacity to answer them now is limited by outdated conceptual approaches to harm, mostly derived from drug evaluation; and by the slow and static nature of traditional research tools. In this article, we propose a re-conceptualizing of the meaning of “harm”, which builds on a global effort focused on health, adding flexibility and richness within a context that acknowledges the physical, mental, and social domains in which it can occur.

## Introduction

In April of 2013, Google announced the Explorer Program, which gave a small group of people the opportunity to use and test the first commercially available wearable computer that enables users to control all of the features of high-end mobile phones and additional augmented reality features using voice commands in natural language [1]. Anticipating a massive public launch of this product in 2014, other players accelerated their efforts to release or sell their own smart glasses, watches, clothes and even wigs. Together with a growing number of commercially available health and fitness monitors, these disruptive technologies promise to accelerate our move into a “hybrid age” [2].

At a time when at least 50% of the population of the world have access to at least one mobile phone [3] and with the number of mobile phone subscriptions expected to exceed the number of humans by the end of 2014, it is essential to ensure that any harm associated with the use of these powerful new devices is anticipated, quickly detected and prevented, or mitigated as much, and as soon as possible.

Questions such as; “Could this be addictive? [4]”, “Could it hurt our children’s brains? [5]”, “Would this be making us dumb? [6]”, “Could this ruin our relationships? [7]”, or “Could this turn us into puppets? [8]”, have been asked before about the technologies that have preceded wearable technologies, from the web to mobile phones. Answering these questions now is urgent as wearable technologies promise to be with us and on us most of the time with the ability to capture information from us, our surroundings, constantly, broadcasting it to others, every instant of our lives.

Any attempt to assess the potentially harmful effects of wearable computers, however, will face two gaping holes in our defensive methodological armor. One of the holes is practical, as our traditional research designs, particularly those belonging to the quantitative realm, are unable to match the speed with which digital technologies are spreading and the dynamic ways in which they are affecting all aspects of our lives [9]. This was evident during the evaluation of the risk of cancer associated with mobile phones, as the validity of the data generated by sophisticated observational studies was threatened by the rapid changes in the technology itself and the way in which patterns of use were evolving during the study period [10]. Now, it would be nearly impossible to design prospective meaningful experiments on the risk of cancer, or any other major potential harmful effects of mobile phones because of the difficulties to find appropriate control groups.

The second hole is conceptual. Even if we had tools with the capacity to match the speed and protean nature of wearable technologies, we will soon realize that our notions of harm are outdated. Criteria that were created when most of the exposures were physical or time-limited, and driven by the need to assess the safety of drugs or invasive therapeutic interventions, can no longer keep up with our need to assess and monitor potentially harmful effects in “real-enough-time”.

Our vocabulary of harm is also very limited when it comes to the richness of the digital world. Words such as “addiction”, “problematic”, “pathological” use, and “disorder” have been used to describe individuals, usually youth, who use the Internet or mobile technology at levels that appear excessive to clinicians [11]. This path, which reflects the medicalization of society that characterized the 20^th^ century, carries the risk of labeling as mental disorders behaviors that may represent “a new normal” [12]. The widely criticized Diagnostic and Statistical Manual of Mental Disorders version 5 (or DSM-5) is of little help, as it only proposes criteria to diagnose “Internet gaming disorder” [13]. This tactic also offers little value because it presupposes the habit-forming properties of the relevant entity. A Medline search of the literature completed in November 2014 does not add much either, as the use of “harm” in combination with terms associated with digital telecommunication technologies failed to identify relevant conceptual frameworks.

This dearth of useful approaches of harm associated with the anticipated wave of wearable devices underscores the urgency with which it is necessary to hold a serious conversation involving clinicians, researchers, policy-makers and the general public. We need to ensure that these technologies can do more good than harm before we step into an era in which ubiquitous computing could not only amplify our human abilities, but also usher in a new way of life [14].

Given that wearable devices have not yet been introduced massively, we still have a cleaner context from which we could draw lessons that might give us new insights in relation to other mobile technologies. This process, unavoidably, must start with a better understanding of the meaning of harm in this new context, while building on the knowledge we have gathered in medicine until now.

## Building on Efforts to Reconceptualize “Health”

### Overview

In December 2008, Alex Jadad and Laura O’Grady issued a call for a global conversation about the meaning of health [15] initiated via a British Medical Journal (BMJ) blog, which grew to include a large number of comments from readers, many of which self-identified as health professionals [16]. As a result of this call to action, the Health Council of the Netherlands hosted a two-day conference attended by a multidisciplinary group of experts to continue the conversation. Guided by a review of the literature, the discussion culminated in a proposed conceptualization of health as the “ability to adapt and to self-manage” in response to physical, mental, or social challenges [17]. As harm is often conceptualized as detracting from health, we might want to build on this work and propose the conceptualization of harm as “any reduction in one’s ability to adapt or to self-manage”, as a result of the use of wearable computers. We could then use this as the foundation for a much wider discussion about harm, not only as it applies to mobile digital telecommunication technologies, but to information and communication technologies in general.

By conceptualizing rather than by trying to define “harm”, as it was the case with “health”, it may be possible to operationalize the term. As it is a dynamic construct, the conceptualization of harm might also add flexibility and richness by being placed within a context that acknowledges the physical, mental, and social domains in which it can occur. Such conceptualization might also enable the incorporation of elements of harm that have been extensively studied in relation to drugs or invasive therapeutic interventions, such as its severity, duration, reversibility, as well as frequency. The following examples attempt to illustrate this.

### Physical Harm

Physical harm corresponds to any physiological or structural dysfunction that could be verified through a biological or medical lens. The main source of concern in this case is the exposure to the electromagnetic waves that all wearable devices emit in the radiofrequency range. Unlike the well-documented effects of ionizing radiation found in X-rays, it has been suggested that the emission of non-ionized radiation by wireless devices could cause genetic and structural cell damage leading to cancer, reproductive defects, neurological degeneration, or immunological disorders [18]. Research to estimate these risks would require studies taking into account that, unlike mobile phones, wearable devices are designed to communicate wirelessly all the time, with much lower levels of radiation emission.

### Psychological Harm

This dimension refers to the lived experience of the users of wearable technologies. Therefore, it encompasses all of the negative emotions, moods, and behaviors that reduce a person’s ability to perform activities of daily living. Examples of potential psychological harm associated with wearable devices include stress in response to a constant barrage of messages requiring immediate attention, or anxiety and confusion from contradictory physiological measures generated by body monitors. Research to understand and describe this type of harm would require a phenomenological approach [19].

### Social Harm

Social harm relates to negative effects of digital telecommunication technologies on communities or groups. This dimension is best understood through a normativist perspective [20], as it is concerned with the norms of goodness and badness in a human collective. Wearable devices, for instance, have the potential to increase harm resulting from privacy violations, cyber-bullying, and abusive handling of personal data by surveillance agencies [21]. These cannot be studied using a medical approach or a phenomenological methodology, because they belong to the cultural, political, and economic realms. Rather, they demand the participation of researchers from the humanities and social sciences.

## What Next?

By conceptualizing rather than by trying to define “harm”, as it is proposed here, it would be possible to operationalize the term as a dynamic construct, with the flexibility and richness needed to acknowledge the physical, mental, and social domains in which it can occur. The proposed conceptualization would also enable the incorporation of elements of harm that have been extensively studied in relation to drugs or invasive therapeutic interventions, such as its severity, duration, reversibility, as well as frequency.

This effort should be taken many steps further. We invite readers to join a global conversation to express their views about our proposal, and to consider supporting the kind of collaborative and iterative processes that are needed to spark and share ideas, which may lead to a better understanding of the potential harm associated with wearable devices.

All it would take to contribute to the conversation. On Twitter, those interested could engage in the conversation by using the hashtag #rethinkingharm and citing the link to this article (which will make the comment appear as tweetation next to this article), or visit the Facebook page, “Rethinking the meaning of harm in the age of wearable technologies.” We also welcome submissions of letters to the editor as well as full articles which propose fresh insights in this area to the “Wearable Devices” section of JMIR mHealth and uHealth.

During the year following the publication of this guest editorial, we will review all of the contributions made by readers to the conversation, using the principles of qualitative content analysis. The meanings of sections of data will be noted using codes that will be developed inductively. Those with similar codes will be grouped into the physical, mental, and social domains of the conceptualization, whenever possible, while those that do not fit will form subcategories, which will be combined to form new categories. All of the responses that lead to an important addition to the conceptualization will be acknowledged. We hope that this call to action will yield many new insights into how we could protect ourselves, and future generations, as we move forward into a new era of human interconnectedness.

## Abbreviations

BMJ: British Medical Journal
DSM-5: Diagnostic and Statistical Manual of Mental Disorders version 5
